# Drivers for low-acuity pediatric emergency department visits in two tertiary hospitals in Switzerland: a cross-sectional, questionnaire-based study

**DOI:** 10.1186/s12913-023-10348-3

**Published:** 2024-01-18

**Authors:** Manon Jaboyedoff, Carl Starvaggi, Joan-Carles Suris, Claudia E. Kuehni, Mario Gehri, Kristina Keitel

**Affiliations:** 1 Department Women-Mother-Child, Service of Pediatrics, Lausanne University Hospital and University of Lausanne, Lausanne, Switzerland; 2 Department of Pediatrics, Division of Pediatric Emergency Medicine, Inselspital, Bern University Hospital, University of Bern, Bern, Switzerland; 3 Institute of Social and Preventive Medicine (ISPM), University of Bern, Bern, Switzerland

**Keywords:** Healthcare use, Low-acuity, Non-urgent, Pediatric emergency department, Socioeconomic status

## Abstract

**Purpose:**

Low-acuity pediatric emergency department (PED) visits are frequent in high-income countries and have a negative impact on patient care at the individual and health system levels. Knowing what drives low-acuity PED visits is crucial to inform adaptations in health care delivery. We aimed to identify factors associated with low-acuity PED visits in Switzerland, including socioeconomic status, demographic features, and medical resources of families.

**Methods:**

We conducted a prospective, questionnaire-based study in the PEDs of two Swiss tertiary care hospitals, Bern and Lausanne. We invited all consecutive children and their caregiver attending the PED during data collection times representative of the overall PED consultation structure (e.g. day/night, weekdays/weekends) to complete a questionnaire on demographic features, socioeconomic status, and medical resources. We collected medical and administrative data about the visit and defined low-acuity visits as those meeting all of the following criteria: (1) triage category 4 or 5 on the Australasian Triage Scale, (2) no imaging or laboratory test performed, and (3) discharge home. We used a binary multiple logistic regression model to identify factors associated with low-acuity visits.

**Results:**

We analysed 778 PED visits (September 2019 to July 2020). Most children visiting our PEDs had a designated primary care provider (92%), with only 6% not having seen them during the last year. Fifty-five per cent of caregivers had asked for medical advice before coming to the PED. The proportion of low-acuity visits was 58%. Low-acuity visits were associated with caregiver's difficulties paying bills (aOR 2.6, 95% CI 1.6 – 4.4), having already visited a PED in the last 6 months (aOR 1.7, 95% CI 1.1 – 2.5) but not with parental education status, nor parental country of birth, parental employment status or absence of family network.

**Conclusion:**

Economic precariousness is an important driver for low-acuity PED visits in Switzerland, a high-income country with compulsory health coverage where most children have a designated primary care provider and a regular pediatric follow-up. Primary care providers and PEDs should screen families for economic precariousness and offer anticipatory guidance and connect those in financial need to social support.

**Supplementary Information:**

The online version contains supplementary material available at 10.1186/s12913-023-10348-3.

## Introduction

Low-acuity pediatric emergency department (PED) visits are frequent in high-income countries [1]. They have a negative impact on patient care and on the healthcare system. At the population level, low-acuity visits increase costs to the healthcare system and contribute to the overcrowding of PEDs [2]. PED overcrowding happens when there is a discrepancy between patient demand and emergency department’s capacity to function efficiently. It increases the risk of overlooking or late diagnosis of critically ill children [3, 4]. At the individual level, low-acuity PED visits put children at risk of superfluous treatments and diagnostic tests, anxiety, and discontinuities in care for those with chronic illnesses [5, 6]. Identifying drivers to low-acuity PED visits is crucial to implement appropriate change in health care delivery. A systematic review of factors that influence families' decision-making for unscheduled pediatric healthcare identified several factors playing a role, such as socioeconomic status, migration, convenience factors (availability of primary-care provider appointment, location of the PED, waiting times), parental perception of urgency and of PED quality of care [7]. The impact of these factors varies depending on the constraints of the healthcare setting in which patients evolve.

We previously conducted a retrospective study including routine data from electronic health records of over more than 50,000 PED visits in two tertiary-care hospitals in Switzerland, and found that 54% of PED visits were low-acuity visits [8]. Low-acuity visits were associated with younger age and convenience factors (proximity of residency and after-hour presentation). However, this retrospective study had not been able to investigate potential low-acuity visit drivers such as socioeconomic status and resources of families visiting our PEDs. Previous studies conducted in Swiss PED focused on parental satisfaction with waiting in time in the PED and on the population of asylum-seekers, but not on low-acuity PED visits [9, 10]. To improve our understanding of factors influencing families to visit PED for low-acuity reasons in Switzerland, we performed this questionnaire-based study. We aimed to assess socioeconomic status, demographic features and medical resources of families visiting PEDs for low-acuity reasons.

## Methods

### Study design and participants

We conducted a cross-sectional, questionnaire-based cohort study in the PED of two tertiary-care hospitals in Switzerland. One hospital is located in a German-speaking region of the country (Bern University Hospital) and the second in a French-speaking region (Lausanne University Hospital). Both PEDs provide care for medical and surgical conditions and are staffed 24/7 by pediatricians and pediatric emergency medicine specialists. They are the only PED in their region, with over 50,000 combined visits per year. Bern PED serves a combined rural and urban population of approximately 400,000 children and cares for around 27,000 PED visits annually. Lausanne PED serves a population of about 320,000 children and cares for over 30,000 visits per year [11, 12].

The study consisted of a patient questionnaire paired with medical data of the PED visit. Patients were included from September 2019 to July 2020, during 1 to 3 random days per week including weekends, and during different hours including nightshifts. This ensured capturing weekly and hourly PED visits variability. All children and adolescents aged 0 to 18 years presenting to one of the participating PED were eligible. A study team member invited all consecutive families to participate in the study while they waited to see the doctor, after having seen a nurse for triage and having filled administrative forms. In the case of life-threatening situations, we approached patients and families at a later stage when the patient's medical condition was stabilized. Approaching families after triage guaranteed no interference or delay in usual care.

Inclusion criteria were being aged 0 to 18 years and visiting Bern or Lausanne PED. Participants were excluded if they did not understand French, German, Italian, English, Albanese, Portuguese, Spanish or Tigrigna or refused to participate in the study.

### Pediatric healthcare in Switzerland and its cost for patients

Pediatricians are the first-line providers of care for children in Switzerland, with nearly 80% of all pre-schoolers regularly seeing a primary care pediatrician [13]. Pediatric emergency departments operating round-the-clock are available in all major cities and freely accessible. All Swiss residents including children must be affiliated to a medical insurance. Premiums are paid by the individuals and are subsidized for the lower-income population. The mean premium for children is 105 CHF per month in 2023 [14, 15]. In addition, patients must contribute to the cost of the healthcare services they receive. This contribution comprises a fixed sum (deductible) and a retention fee of 10% of any additional healthcare expenses. The deductible is variable between 0 and 600 CHF and is chosen by the patient. The higher the deductible is, the lower the monthly premiums are. The maximum retention fee for children is 350 CHF per year, while the median monthly disposable income is 3,930 CHF [16].

### Outcome and low-acuity definition

Acuity of PED visit was the outcome of our study. There is no consensus on the definition of level of acuity of PED visits in the literature [1]. Triage alone is often used as a proxy for acuity, but some studies used either one or a combination of criteria including triage level, diagnosis, resources used, and disposition. We chose to define acuity as a combination of urgency and complexity of the PED visit. We used triage level to measure urgency of the PED visit and use of medical resources and disposition to measure complexity. Thus, we defined low-acuity visits as those meeting all of the following criteria: i) triage level 4 or 5 on the Australasian Triage Scale (ATS) [17], ii) neither imaging nor laboratory testing performed, and iii) discharge home. PED visits not meeting all those criteria were classified as high-acuity.

### Explanatory variables and questionnaire

We considered demographic features, socioeconomic status and medical resources as potential determinants of low-acuity PED visits and included these features in a questionnaire (S-Fig. 1). Demographic and socioeconomic questions were adapted from the Swiss Structural Survey, a component of the Population Census providing information on population, households, families, employment, mobility, education, language and religion which is developed by the Swiss Federal Statistical Office [18]. For economic status, we included the question "Did you have difficulties paying your household bills during the last 12 months?" which is an effective way of screening for financial vulnerability in Switzerland [19]. Questions relating to medical resources were developed by the research team specifically for the purpose of this study. They included whether participants had asked for medical advice before visiting the PED (“Before deciding to come to the Pediatric Emergency Ward, did you ask for medical advice within the past 24 h?”). The questionnaire was translated into 3 national languages (French, German and Italian), into English and into the 4 most frequent languages spoken by the immigrant population (Albanese, Portuguese, Spanish and Tigrigna). Questionnaires were collected and managed using REDCap electronic data capture tools hosted at Lausanne University Hospital [20, 21]. REDCap (Research Electronic Data Capture) is a secure, web-based software platform that supports data capture for research studies. Participants filled out the questionnaire directly on a computer or tablet while waiting in the PED waiting room. After the PED visit, a study team member extracted medical data from the electronic health records. Data retrieved from electronic health records included triage scale, diagnostic tests (imaging and laboratory studies), discharge diagnosis and disposition. Discharge diagnoses were grouped according to the grouping system for child ED visits of the Pediatric Emergency Care Applied Research Network (PECARN) [22].

### Statistical analysis

We described study population characteristics using median and interquartile range (IQR) for continuous variables and number and proportion for categorical variables. We compared characteristics of low-acuity and high-acuity visits using Pearson's Chi-square test. To identify factors associated with low-acuity visits, all statistically significant variables p < 0.05 in univariable analysis were included in a binary multiple logistic regression model including baseline and socioeconomic characteristics: gender of the child, age of the child and of the parents, parental migration, education, and employment status. We aimed to evaluate the association of each of these socioeconomic components with low-acuity PED visits. Missing data were excluded from the analysis. We analysed the data using STATA (StataCorp. 2019. Stata Statistical Software: Release 16. College Station, TX: StataCorp LLC).

### Ethics

This study was approved by the Cantonal Research Ethics Committees of Cantons of Vaud and Bern (project number 2019–00538). This study was conducted in accordance with the ethical standards of both Ethics Committees and with the principles of the Declaration of Helsinki. Informed consent was obtained from the legal guardian at the time of PED visit for all participants.

## Results

### Study population characteristics

Nine hundred ninety-eight patients were assessed for eligibility. One hundred ninety-three refused to participate. The participation rate was 78%. Three patients were excluded because their visit to the PED had been scheduled, and 24 were excluded because the outcome data were missing. We thus analyzed 778 visits, 401 from Lausanne and 377 from Bern (flow diagram, S-Fig. 2).

The median age of patients was 5 years (IQR 1 – 10) and 50% were female. Most children (95%) were accompanied to the PED by their mother, father or both, while the rest were accompanied by a member of the family (grand-parent, uncle or aunt, sibling), a person employed to take care of the child, a friend, or other.Most visits (87%) had a low-urgency triage (ATS 4 and 5). Imaging studies were performed for 26% of visits and laboratory studies for 15%. Nine per cent of the visits led to hospital admission. Four hundred fifty-two amongst 778 PED visits met our criteria for low-acuity visits, representing 58% of the study population. The most frequent discharge diagnoses groups were trauma, ENT diseases, gastro-intestinal diseases, systemic states and respiratory diseases (S**-**Table 1). Overall, the sample population had similar characteristics as the entirety of PED visits during the same time period (Table 1).

**Table 1:** Characteristics of the study population

Characteristics	Study participants *N* = 778		Total PED visits during the study period^a^ *N* = 46,340	
Age: < 1 month	3	(0%)	508	(1%)
Age: 1 – 11 months	94	(12%)	6136	(13%)
Age: 12 – 23 months	108	(14%)	5901	(13%)
Age: 2 – 5 years	216	(28%)	13,832	(30%)
Age: 6 – 11 years	212	(27%)	12,200	(26%)
Age: 12 – 17 years	145	(19%)	7763	(17%)
Gender: Female	388	(50%)	21,080	(45%)
Accompanied by parent(s)	731	(95%)	NA	NA
ATS 1	2	(0%)	216	(0%)
ATS 2	27	(3%)	2674	(6%)
ATS 3	76	(10%)	6861	(15%)
ATS 4	257	(33%)	12,222	(26%)
ATS 5	416	(54%)	24,214	(52%)
Imaging performed	200	(26%)	8945	(19%)
Laboratory study performed	118	(15%)	9434	(20%)
Admissions	67	(9%)	4863	(10%)

*PED* Paediatric Emergency Department, *ATS* Australasian Triage Scale

Values are median number (%)

^a^Missing ATS data for 153 PED visits

### Demographic, socioeconomic characteristics and medical resources

Fifty-three per cent of the children visiting our PED's were a first child and 25% only child. Most children (63%) had at least one parent (mother or father) with a higher education degree. A third had at least one parent unemployed, and 17% had both of their parents working full-time. Most children (89%) were born in Switzerland, but 41% had at least one parent born abroad. In 17% of children, both parents did not have extended family in Switzerland. Twenty percent of families declared having difficulties paying household bills (Table 2).

**Table 2:** Demographic and socio-economic characteristics of the study population in relation with PED visit acuity

Characteristics	Number of observations available	All PED visits *N* = 778		Low-acuity PED visits *N* = 452		High-acuity PED visits *N* = 326		*p*-value†
Age ≤ 5 years	778	374	(48%)	241	(53%)	133	(41%)	0.001
Gender: Female	776	388	(50%)	218	(48%)	170	(52%)	0.275
First child	636	339	(53%)	209	(52%)	130	(56%)	0.385
Only child	726	180	(25%)	112	(26%)	68	(23%)	0.324
Any parent < 26 years old	752	35	(5%)	26	(6%)	9	(3%)	0.047
Mother without education or mandatory education only	711	105	(15%)	67	(16%)	38	(13%)	0.209
Father without education or mandatory education only	703	98	(14%)	69	(17%)	29	(10%)	0.008
Mother unemployed	732	187	(26%)	121	(29%)	66	(22%)	0.036
Father unemployed	716	47	(7%)	32	(8%)	15	(5%)	0.140
Child born in Switzerland	752	671	(89%)	388	(89%)	283	(90%)	0.490
Mother born abroad	744	386	(52%)	246	(57%)	140	(45%)	0.001
Father born abroad	727	370	(51%)	235	(56%)	135	(44%)	0.003
Both parents without family in Switzerland	727	124	(17%)	80	(19%)	44	(15%)	0.133
Difficulties to pay household bills	703	141	(20%)	102	(25%)	39	(13%)	< 0.001
Travel time to PED < 15 min	766	299	(39%)	197	(44%)	102	(32%)	0.001

*PED* Paediatric emergency department

Values are number (%). For proportion, the denominator is the number of observations available

Definition of low-acuity PED visits: Triage ATS 4 or 5, no laboratory nor imaging tests and no hospital admission

†*p*-value for comparison of acuity. Pearson’s χ2 test

Most children visiting our PEDs had a dedicated primary care provider (92%), and only 6% had not seen him/her during the last year. Thirty-five percent of children had already visited a PED in the last 6 months. Fifty-five percent of caregivers (i.e., the person taking care of the child) accompanying the children to the PED had asked for medical advice before coming to the PED (Table 3).

**Table 3:** Medical resources of the study population in relation with PED visit acuity

**Medical resources**	**Number of observations available**	**All PED visits** ***N*** = 778		**Low-acuity PED visits** ***N*** = 452		**High-acuity PED visits** ***N*** = 326		***p*-value**†
Has a paediatrician or GP	756	698	(92%)	414	(93%)	284	(91%)	0.167
Paediatrician or GP not seen during the last year	645	38	(6%)	20	(5%)	18	(7%)	0.309
Asked for medical advice before coming to PED	638	350	(55%)	187	(50%)	163	(62%)	0.002
Already visited a PED in the last 6 months	744	275	(37%)	186	(43%)	89	(29%)	< 0.001

*PED* Paediatric emergency department, *GP* General practitioner

Values expressed as number (%). For proportion, the denominator is the number of observations available

Definition of low-acuity PED visits: Triage ATS 4 or 5, no laboratory nor imaging tests and no hospital admission

^†^*p*-value for comparison of acuity. Pearson’s χ2 test

### Factors associated with low-acuity PED visits

In the univariable analysis, child factor associated with low-acuity visits was being younger than 5 years. Parental factors associated with low-acuity visits were young parental age (< 26 years), maternal unemployment, paternal lower education status and parental foreign country of birth. Household factors associated with low-acuity visits were difficulties to pay household bills, PED proximity and having already visited the PED in the last 6 months. Asking for medical advice before visiting the PED was negatively associated with low-acuity. Factors not associated with PED visit acuity in the univariable analysis were: Child’s country of birth, being an only child or a first child, maternal education status, paternal employment status and absence of extended family (Tables 2 and 3).

In the multivariable analysis, the adjusted odds (aOR) of a PED visit to be a low-acuity one were 2.6 (95% CI 1.6 – 4.4) times higher when the caregiver had difficulties paying household bills and 1.8 (95% CI 1.2 – 2.7) times higher when the child had already visited a PED in the last 6 months. There was a trend towards age younger than 5 years and PED proximity being associated with low-acuity visits (aOR 1.4 95% CI 1.0 – 2.0 and aOR 1.5 95% CI 1.0 – 2.1). Parental age, place of birth of the child or of the parents, parental education, parental employment status, medical advice before PED visit and travel time to PED were not associated with acuity of the visit in the multivariable analysis (Table 4).

**Table 4:** Logistic regression model of characteristics associated with low-acuity PED visits

Characteristics	Adjusted Odds Ratio (95% CI)	
Age ≤ 5 years	1.4	(1.0 – 2.0)
Gender: female	0.8	(0.6 – 1.2)
Any parent < 26 years old	1.2	(0.4 – 3.3)
Child born in Switzerland	0.8	(0.4 – 1.6)
Mother born abroad	1.5	(0.9 – 2.3)
Father born abroad	1.0	(0.6 – 1.6)
Difficulties to pay household bills	2.6	(1.6 – 4.4)
Mother without education or mandatory education only	0.7	(0.4 – 1.5)
Father without education or mandatory education only	1.2	(0.6 – 2.5)
Mother unemployed	0.9	(0.6 – 1.4)
Father unemployed	0.7	(0.3– 1.6)
Asked for medical advice before coming to PED	0.7	(0.5 – 1.1)
Already visited a PED in the last 6 months	1.7	(1.1 – 2.5)
Travel time to PED < 15 min	1.5	(1.0 – 2.1)

*PED* Paediatric emergency department

Definition of low-acuity PED visits: Triage ATS 4 or 5, no laboratory nor imaging tests and no hospital admission

To investigate the reasons why having difficulties paying bills is associated with low-acuity visits, we performed a sensitivity analysis to explore an association between difficulties paying bills and reasons to come to the PED (Table 5). Difficulties paying bills was associated with caregiver declaring not being able to visit the pediatrician within opening hours and declaring that their child’s problem was too serious.

**Table 5:** Sensitivity analysis: Difficulties paying bills and reasons to come to the emergency ward

	**Difficulties paying households bills in the last 12 months**				
Why did you come to the emergency ward?	**Yes** ***N*** = 141		**No** ***N*** = 562		***p*-value**
My child’s problem was too serious	52	(37%)	156	(28%)	0.034
The Pediatric Emergency ward was the best place for my child’s medical problem	39	(28%)	144	(26%)	0.622
It would take too long to get an appointment with the doctor	24	(17%)	73	(13%)	0.215
I can’t visit my doctor within opening hours	14	(10%)	28	(5%)	0.027
It is close to my home	1	(1%)	9	(2%)	0.424
I did not think of it	1	(1%)	2	(0%)	0.565
I have been referred by a healthcare professional	28	(20%)	154	(28%)	0.067
Other	17	(12%)	53	(10%)	0.352
I do not know or do not want to answer	0	(0%)	4	(1%)	0.315

Values expressed as number (%)

^†^*p*-value for comparison of difficulties paying bills. Pearson’s χ2 test

## Discussion

We found that economic precariousness is associated with low-acuity PED visits, but not immigration, employment, or education status of the family. To our knowledge, this is the first study exploring socioeconomic and demographic drivers for low-acuity PED visits in Switzerland.

### Economic precariousness as a driver for low-acuity PED visits in Switzerland

The main driver for low-acuity PED visits in our study population was economic precariousness, after adjusting for other demographic and socioeconomic characteristics. Switzerland is a high-income country with one of the world's highest gross domestic products (GDP) per capita and one of the highest standards of living in Europe [23, 24]. Poverty threshold is defined by an income below 60% of the median disposable income by the European Union, corresponding to 2279 CHF for a single person. In Switzerland, 9% of the population is affected by income poverty [25]. Medical insurance is compulsory in Switzerland, but medical care requires substantial payment from patients. Patients pay insurance premiums, up to 600 CHF of deductibles per year for children and 10% of healthcare expenses. These expenses are partly subsidized for the lower-income population, but poverty status impacts healthcare use in Switzerland. Indeed, several studies reported a high prevalence of healthcare renunciation for economic reasons in the low-income adult Swiss population [26, 27]. In Swiss children, a study reported a higher prevalence of caries in those from a lower socioeconomic background – dental care not being covered by medical insurance [28]. Children growing up in families in financial distress may underutilize primary care, leading to more frequent exacerbation and suboptimal management of chronic illnesses like asthma [29]. Per capita out-of-pocket healthcare costs in Switzerland are almost three times as high as the Organisation for Economic Cooperation and Development (OECD) average, and 6% of household with children have payment arrears of healthcare insurance premiums [30, 31]. In Switzerland, children usually have a co-payment of around 10% regardless of the type of visit (primary care or PED visit). PED visits are often charged more than a planned visit to a primary care provider and hence are more expensive for families. There are some hypothesis why families facing economic difficulties choose to visit the PED for low-acuity reasons even if the co-payment is often higher for a PED visit. First, the billing process differs between hospital and private practices: for consultations with primary care providers, parents often have to pay the bill first and request reimbursement from the insurance afterwards. Bills from the PED are usually paid directly by the insurance company (except for the co-payment). Families with economic difficulties may therefore choose to visit the hospital to avoid facing payment issues with their own primary care provider, fearing that the doctor-patient relationship could be damaged. Also, parent may not be aware that an ED visit usually results in higher costs than a visit to their pediatrician. Studies conducted in other settings found that healthcare costs played a role in healthcare system use in the low-income population, for example in the adult population in the USA [32, 33]. In a context similar to ours, an Australian study – a country with universal health insurance – showed that lower-income families were more likely to declare that they would attend the PED if a primary care visit incurred a co-payment of $7 [34]. Second, visits to a primary care provider occur during office hours, which means that working caregivers must take time off work to bring their child to the pediatrician. In our study, caregivers having difficulties paying bills were more likely to declare not being able to visit the PED during opening hours. In Switzerland, workers are allowed to take 3 days' paid leave to look after their child per sickness, but it is not the case for precarious workers like those hourly-paid and might not always be readily accepted by the employer, implying a loss of salary.

Previous studies reported a significant impact of socioeconomic status on PED use. One of these studies conducted in the USA on the impact of Child Opportunity Index (COI) – a measure of structural neighborhood context using indicators in the education, health and environment, and social and economic domains – found that children from neighborhoods with low COI had higher PED use and more frequent low-resource PED visits [35]. Data from European countries with national health insurance point towards the same findings [36, 37]. A study conducted in the UK showed that the most deprived children were more likely to visit the PED than the least deprived [38, 39].

Migration was not associated with low-acuity PED visits in our studies. These findings are consistent with another Swiss study that found comparable hospital admissions for ambulatory-care-sensitive conditions between asylum-seeking and non-asylum-seeking children and with a cohort study conducted in the UK on PED utilization rates and maternal migration status [10, 40].

In our population, a child having already visited a PED in the previous 6 months was more likely to visit for a low-acuity reason, which might reflect repetitive healthcare-seeking behaviors. Although not significant, there was a trend toward age younger than 5 years and PED proximity being associated with low-acuity visits. These two factors had also been associated with low-acuity visits in our retrospective study including more than 50,000 PED visits and it is likely that these factors would have been significant in this study had we had with a larger sample [8].

Our findings confirm that most children have a regular primary care provider in Switzerland (92% of our study population) and that they see him/her regularly. We also found that more than half (55%) of caregivers had searched for medical advice before coming to the PED. Those who did ask for medical advice (doctor or medical hotline) prior to the visit were not less likely to visit the PED for a low-acuity reason than those who did not. Therefore, the reason why families choose to visit the PED for low-acuity reasons seems to be unrelated to primary care follow-up.

### Screening for and addressing economic precariousness during child health encounters

Systematic screening of social needs, including economic precariousness, is usually considered as a component of primary care [41, 42]. Randomized controlled trials showed that systematic screening and referral for social determinants during well-child visits and urgent-care could decrease social needs and improve receipt of community resources and parent-reported child health [43, 44]. While pediatricians might not be trained to address financial needs of families, they play a key role in screening for economic precariousness and in connecting families with social support. Social interventions should therefore be incorporated in pediatric healthcare delivery.

### Strengths and limitations

The strengths of this study include the granularity of the data, covering many demographic and socioeconomic aspects and allowing a comprehensive definition of PED visit acuity. We strived to be representative of the population visiting our PEDs located in two of the five Swiss tertiary-care pediatric hospitals, in two different linguistic regions of Switzerland. We included patients at all hours and on all days, and translated our questionnaire into the 8 most spoken languages in Switzerland. Unlike other studies on low-acuity PED visits, we included high-acuity PED visits as well, allowing us to be representative of the general population visiting the PEDs.

Our study has some limitations. It started before the COVID19 pandemic and had to be interrupted during the first months of the pandemic. PED visit patterns changed during the pandemic and may not have returned to baseline [45]. It was also conducted in a tertiary-care setting, and its results might not be generalizable to populations living closer to non-academic regional PEDs or to other countries with different social security and healthcare systems. Although we identified that financial difficulties are associated with low-acuity PED visits in our setting (high-income country with compulsory medical insurance), our study is not able to identify the root causes of this association.

## Conclusions

Economic precariousness is an important driver for low-acuity PED visits in Switzerland, a high-income country with compulsory health coverage where most children have a designated primary care provider and a regular pediatric follow-up. These findings are important to guide the design of health policies aiming to optimize child health and lower unnecessary PED visits. Public health authorities, primary care providers and PEDs should work on a better integration of social services in pediatric healthcare delivery. Future research is needed to identify the root causes of the association between economic precariousness and low-acuity PED visits in Switzerland.

### Supplementary Information

**Additional file 1:** **Figure S1. **Questionnaire. **Figure S2.** Flow diagram of PED visits included.

## Data Availability

The datasets presented in this article are not readily available because they contain patient sensitive data. Requests to access the datasets should be directed to the corresponding author.

## Abbreviation

PED: Pediatric emergency department
