# Supplementary material for: Drivers for low-acuity pediatric emergency department visits in two tertiary hospitals in Switzerland: a cross-sectional, questionnaire-based study
Source: BMC Health Serv Res. 2024 Jan 18;24:103. doi: 10.1186/s12913-023-10348-3 (PMC10797974; doi:10.1186/s12913-023-10348-3)
Supplement: Supplementary file 1 — Additional file 1: Figure S1. Questionnaire. Figure S2. Flow diagram of PED visits included. [file 12913_2023_10348_MOESM1_ESM.pdf]

Drivers for low-acuity pediatric emergency department consultations in two tertiary hospitals in Switzerland: a prospective, questionnaire-based study

Manon Jaboyedoff, Carl Starvaggi, Joan-Carles Suris, Claudia E Kuehni, Mario Gehri, Kristina Keitel

Supplementary information

S-figure 1: Questionnaire

Evaluation of factors influencing the use of paediatric emergency department in non-urgent situations: Patient questionnaire English version

1. What is the main problem that brought you to the Pediatric Emergency Ward today?

2. How did you and your child arrive today?

- By car
- Walking
- By taxi
- By public transport
- By ambulance
- By bike
- Other
- I do not know or do not want to answer

2b Other: Please specify

3. How long did it take for you to get to the Pediatric Emergency Ward today?

- Less than 15 minutes
- 15 - 30 minutes
- 30 minutes - 1 hour
- More than 1 hour
- I do not know or do not want to answer

4. Where did you come from today?

- From home
- From school, nursery or daycare
- From a Doctor's office
- From another hospital
- Other
- I do not know or do not want to answer

4b Other: Please specify

5. What is your relationship to the child?

- I am the Patient
- Mother
- Father
- Mother-in-law
- Father-in-law
- Grand-parent
- Aunt or uncle
- Sibling
- Nanny/Sitter
- Friend
- Other
- I do not know or do not want to answer

5b Other: please specify

6.1 Does the child have a regular doctor?

- Yes: a pediatrician
- Yes: a general practitioner
- No
- I do not know or do not want to answer

6.2 When did your child see her/him last?

- Within the last 2 days
- Within the last 7 days
- Within the last 30 days
- Within the last 6 months
- Within the last year
- More than a year ago
- I do not know or do not want to answer

7 Has your child been referred to the pediatric emergency by a healthcare professional?

- Yes
- No

7.1.1 Who referred the child to the Notfallzentrum für Kinder und Jugendliche?

- Doctor
- Kispiphone
- Medphone
- Insurance medical hotline
- An other hospital
- Other
- I do not know or do not want to answer

7.1.1 Other: please specify

7.1 Before deciding to come to the Pediatric Emergency Ward, did you ask for medical advice within the past 24h?

- No
- Yes: the doctor by phone
- Yes: the doctor seen in consultation
- Yes: the telephone hotline of the on duty doctors
- Yes: insurance medical hotline
- Yes: another hospital
- Yes: other
- I do not know or do not want to answer

7.1.1 Other: please specify

7.1.2 Were you advised to go to the Emergency Ward?

- Yes
- No
- I do not know or do not want to answer

7.2 Why did you come to the emergency ward?

- My child's problem was too serious
- The Pediatric Emergency Ward was the best place for my child's medical problem
- It would take too long to get an appointment with the doctor

- I can't visit my doctor within opening hours
- It is close to my home
- I did not think of it
- I have been referred by a healthcare professional
- Other
- I do not know or do not want to answer

7.2.1 Other: please specify

8.1 Before deciding to come to Pediatric Emergency Ward, did you search for information on the Internet?

- Yes
- No
- I do not know or do not want to answer

8.1.2 Which website(s) did you visit?

8.2 Before deciding to come to Pediatric Emergency Ward, did you look for information on a smartphone app?

- Yes
- No
- I do not know or do not want to answer

8.2.1 Which app(s) did you use?

9 How many times in the past 6 months has your child visited any Emergency Ward?

10 When you decided to come to the Emergency Ward, how soon did you expect to be seen by a doctor?

- Immediately
- Within 30 minutes
- Within 1 hour
- Within 2 hours
- Within 4 hours
- More than 4 hours
- I do not know or do not want to answer

11 How would you rate the severity of your child's condition today?

- Mild
- Moderate
- Severe
- I do not know or do not want to answer

12 Where was your child born?

- In Switzerland
- Abroad
- I do not know or do not want to answer

12b Pays de naissance de l'enfant / Geburtsland des Kindes / Country of birth of the child

13 Where was the child's mother born?

- In Switzerland
- Abroad
- I do not know or do not want to answer

13b Pays de naissance de la mère / Geburtsland der Mutter / Country of birth of the mother

14 What is the age of the mother of the child?

- Less than 20 year old
- 20 - 25 years old
- 26 - 30 years old
- 31 - 35 year old
- 36 - 40 years old
- 41 - 45 years old
- More than 45 years old
- I do not know or do not want to answer

15 Where was the child's father born?

- In Switzerland
- Abroad
- I do not know or do not want to answer

15b Pays de naissance du père / Geburtsland des Vaters / Country of birth of the father

16 What is the age of the father of the child?

- Less than 20 year old
- 20 - 25 years old
- 26 - 30 years old
- 31 - 35 year old
- 36 - 40 years old
- 41 - 45 years old
- More than 45 years old
- I do not know or do not want to answer

17 With whom does the child live most of the time?

- Both parents
- Mother
- Father
- Mother half of the time, father half of the time
- Other
- I do not know or do not want to answer

17b Other: please specify

18 How many siblings and step- or half-siblings does the child have?

19 Where does the child rank among his/her siblings?

- 1st child
- 2nd child
- 3rd child
- 4th child
- 5th child
- 6th child and more

20 Do family members of the mother (her own parents or siblings) live in Switzerland?

- Yes
- No
- I do not know or do not want to answer

21 Do family members of the father (his own parents or siblings) live in Switzerland?

- Yes
- No
- I do not know or do not want to answer

22 What is the highest education degree achieved by the mother of the child?

- Did not go to school
- Mandatory education (or a few years at school)
- Apprenticeship
- Higher technical or commercial college
- University
- I do not know or do not want to answer

23 What is the highest education degree achieved by the father of the child?

- Did not go to school
- Mandatory education (or a few years at school)
- Apprenticeship
- Higher technical or commercial college
- University
- I do not know or do not want to answer

24 What is mother's current work situation?

- Full-time employment (including apprenticeship)
- Part-time employment (including apprenticeship)
- Non-employed
- Stay-at-home mom
- I do not know or do not want to answer

25 What is father's current work situation?

- Full-time employment (including apprenticeship)
- Part-time employment (including apprenticeship)
- Non-employed
- Stay-at-home dad
- I do not know or do not want to answer

26 Did you have difficulties paying your household bills during the last 12 months?

- Yes
- No
- I do not know or do not want to answer

27 Compared to other families in Switzerland, the financial situation of your family is...

- Above average
- Average
- Below average
- I do not know or do not want to answer

28 Do you have any further comments about this topic or about l'Hôpital de l'Enfance / Notfallzentrum für Kinder und Jugendliche?

Drivers for low-acuity pediatric emergency department consultations in two tertiary hospitals in Switzerland: a prospective, questionnaire-based study

Manon Jaboyedoff, Carl Starvaggi, Joan-Carles Suris, Claudia E Kuehni, Mario Gehri, Kristina Keitel

S-figure 2: Flow diagram of PED visits included

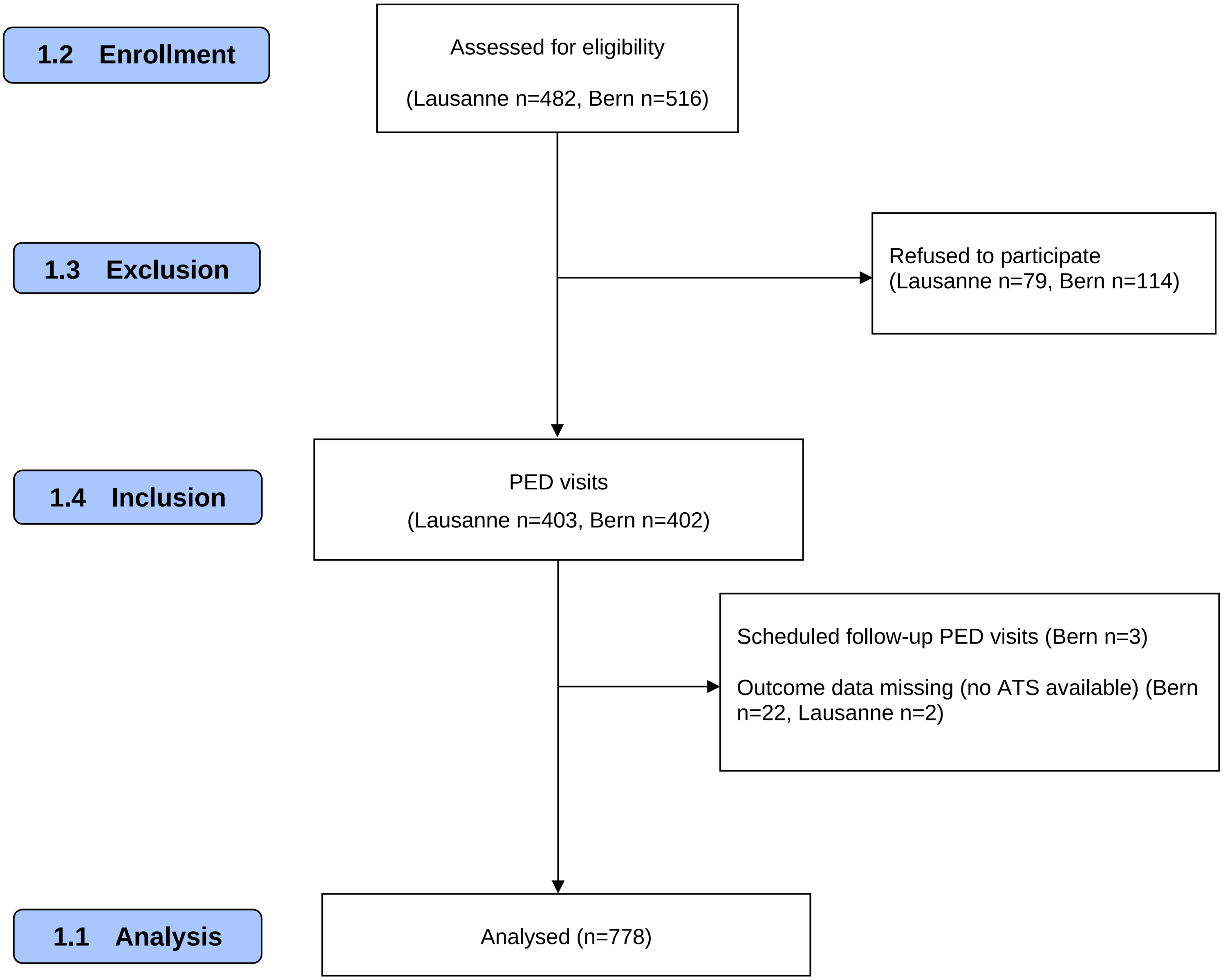

S-table 1: PED visits diagnoses

PED: Paediatric Emergency Department

Diagnosis grouping system according to Alessandrini et al. Acad Emerg Med. 2010.

Diagnosis at the end of PED visit	All PED visits N = 778		Low-acuity PED visits N = 452		High-acuity PED visits N = 326	
**Trauma**	281	(36%)	129	(29%)	152	(47%)
**ENT, dental, and mouth diseases**	135	(17%)	115	(25%)	20	(6%)
**Gastrointestinal diseases**	85	(11%)	51	(11%)	34	(10%)
**Systemic states**	57	(7%)	25	(6%)	32	(10%)
**Respiratory diseases**	45	(6%)	28	(6%)	17	(5%)
**Skin, dermatologic, and soft tissue diseases**	36	(5%)	29	(6%)	7	(2%)
**Neurologic diseases**	30	(4%)	13	(3%)	17	(5%)
**Musculoskeletal and connective tissue diseases**	26	(3%)	9	(2%)	17	(5%)
**Urinary tract diseases**	12	(2%)	2	(0%)	10	(3%)
**Eye diseases**	9	(1%)	9	(2%)	0	(0%)
**Genital and reproductive diseases**	9	(1%)	5	(1%)	4	(1%)
**Other**	5	(1%)	2	(0%)	3	(1%)
**Psychiatric, behavior, and substance abuse**	5	(1%)	5	(1%)	0	(0%)
**Hematologic diseases**	3	(0%)	0	(0%)	3	(1%)
**Toxicologic emergencies (including environmental)**	3	(0%)	0	(0%)	3	(1%)
**Child abuse**	2	(0%)	2	(0%)	0	(0%)
**Circulatory and cardiovascular diseases**	2	(0%)	2	(0%)	0	(0%)
**Fluid and electrolyte disorders**	2	(0%)	0	(0%)	2	(1%)
**Neoplastic diseases (cancer, not benign neoplasms)**	2	(0%)	0	(0%)	2	(1%)
**Missing**	29	(4%)	26	(6%)	3	(1%)

Definition of low-acuity visits: Triage ATS 4 or 5, no laboratory nor imaging tests and no hospital admission
